# Long term outcomes of patients with chronic kidney disease after COVID-19 in an urban population in the Bronx

**DOI:** 10.1038/s41598-025-90153-6

**Published:** 2025-02-19

**Authors:** Jason Y. Lu, Justin Y. Lu, Stephen Wang, Katie S. Duong, Sonya Henry, Molly C. Fisher, Tim Q. Duong

**Affiliations:** 1 Department of Radiology, Albert Einstein College of Medicine and Montefiore Medical Center, Bronx, NY USA; 2 Department of Surgery, Beth Israel Deaconess Medical Centerand, Harvard Medical School, Boston, MA USA; 3 Department of Medicine, Nephrology Division, Albert Einstein College of Medicine, Bronx, NY USA

**Keywords:** Long covid, Post-acute sequela of COVID-19 (PASC), Kidney disorders, Creatinine, EGFR, Dialysis, Nephrology, Epidemiology

## Abstract

We investigated the long-term kidney and cardiovascular outcomes of patients with chronic kidney disease (CKD) after COVID-19. Our retrospective cohort consisted of 834 CKD patients with COVID-19 and 6,167 CKD patients without COVID-19 between 3/11/2020 to 7/1/2023. Multivariate competing risk regression models were used to estimate risk (as adjusted hazard ratios (aHR) with 95% confidence intervals (CI)) of CKD progression to a more advanced stage (Stage 4 or 5) and major adverse kidney events (MAKE), and risk of major adverse cardiovascular events (MACE) at 6-, 12-, and 24-month follow up. Hospitalized COVID-19 patients at 12 and 24 months (aHR 1.62 95% CI[1.24,2.13] and 1.76 [1.30, 2.40], respectively), but not non-hospitalized COVID-19 patients, were at higher risk of CKD progression compared to those without COVID-19. Both hospitalized and non-hospitalized COVID-19 patients were at higher risk of MAKE at 6-, 12- and 24-months compared to those without COVID-19. Hospitalized COVID-19 patients at 6-, 12- and 24-months (aHR 1.73 [1.21, 2.50], 1.77 [1.34, 2.33], and 1.31 [1.05, 1.64], respectively), but not non-hospitalized COVID-19 patients, were at higher risk of MACE compared to those without COVID-19. COVID-19 increases the risk of long-term CKD progression and cardiovascular events in patients with CKD. These findings highlight the need for close follow up care and therapies that slow CKD progression in this high-risk subgroup.

## Introduction

Patients with chronic kidney disease (CKD) are at significantly higher risk of adverse outcomes after COVID-19, including increased rates of hospitalization, critical illness, and mortality compared to those without CKD^1,2^. This increased vulnerability is due to several factors associated with both CKD and COVID-19. CKD is characterized by immune system dysfunction due to circulating uremic toxins and increased inflammation, which impairs the body’s ability to respond to stress and increases susceptibility to severe infections^3^. Similarly, severe COVID-19 also causes systemic inflammation and release of pro-inflammatory cytokines, which promote organ injury^4^. Dysregulated immune responses because of severe COVID-19 may also lead to prolonged illness.

Acute kidney injury (AKI) is common in severe COVID-19 and patients with CKD are at increased risk of AKI compared to those without CKD. Observational studies have estimated that 15–48% of individuals with COVID-19 associated AKI have underlying CKD^5–8^. Conversely, consequences of AKI include permanent loss of kidney function, development or progression of CKD. A multicenter study of 235 patients with CKD who developed hospital AKI observed that nearly 30% developed end stage kidney disease (ESKD) requiring dialysis over 5 year follow up^9^.

Patients with CKD are also at increased risk of major adverse cardiovascular events (MACE) and major adverse kidney events (MAKE) compared to the general population^10–12^. CKD promotes hypertension, dyslipidemia, and vascular calcification, which increases the risk of adverse cardiovascular events such as myocardial infarction and stroke. Additionally, underlying cardiovascular disease is a risk factor for CKD progression due to reduced blood flow to the kidneys, causing ischemic injury.

Although acute outcomes of patients with CKD and COVID-19 have been well-described^13–16^, long-term kidney and cardiovascular outcomes among COVID-19 patients with CKD have not been fully elucidated. For the reasons stated above, this subgroup may be at increased risk of accelerated kidney function decline and major adverse kidney and cardiovascular events long after resolution of SARS-CoV-2 infection^17,18^.

The objective of our study was to assess the long-term outcomes of patients with CKD up to 24 months after COVID-19 compared to patients with CKD without a diagnosis of COVID-19. Specifically, we evaluated the risk of CKD progression to a more advanced stage and the risk of major adverse kidney events (MAKE) and major adverse cardiovascular events (MACE).

## Materials and methods

### Data source and extraction

This study was approved by the Einstein-Montefiore Institutional Review Board (#2021–13,658) with an exemption for informed consent and a Health Insurance Portability and Accountability Act (HIPPA) waiver. The Montefiore Health System serves a large low-income, racially, and ethnically diverse population. Data originated from the Montefiore Health System consists of multiple hospitals and outpatient clinics located in the Bronx and its environs. De-identified health data were made available for research after standardization to the Observational Medical Outcomes Partnership (OMOP) Common Data Model (CDM) version 6. OMOP CDM represents healthcare data from diverse sources, which are stored in standard vocabulary concepts. This approach allows for the systematic analysis of disparate observational databases, including data from the electronic medical record (EMR), administrative claims, and disease classifications systems (e.g., ICD-10, SNOWMED, LOINC, etc.). Data were subsequently exported and queried as SQLite database files using the DB Browser for SQLite (version 3.12.0). To ensure data accuracy, our team performed extensive cross validation of all major variables extracted by manual chart reviews on subsets of patients. All methods were carried out in accordance with relevant guidelines and regulations, including those of stated in the “Declaration of Helsinki.” Studies using an earlier version of this large database to address different questions have been reported^19–34^.

The date of a patient’s first positive COVID-19 result or the first visit to the Montefiore Health System after March 1st, 2020 was used as the index date for the COVID-19 and non-COVID-19 cohorts, respectively. We excluded patients with preexisting end-stage kidney disease (ESKD) requiring dialysis, those with a previous eGFR less than 15 mL/min/1.73m^2^, those without outpatient creatinine values before and after index date, and those who did not meet our CKD definition.

Demographics and clinical comorbidities were extracted from electronic medical records. Demographic data included age, sex, ethnicity, and race. Chronic comorbidities included hypertension, diabetes, chronic obstructive pulmonary disease (COPD), asthma, liver disease, smoking status, heart failure, and cancer. Hospitalization status within two weeks of index date, critical illness (defined by initiation of invasive mechanical ventilation or ICU admission within two weeks of index date), and post COVID-19 mortality were also extracted. Acute kidney injury (AKI) was defined as a 1.5-fold increase in serum creatinine from baseline^35,36^. Creatinine values from 6 days prior to 30 days after COVID diagnosis or index date were used to define AKI.

### Exposure variable

The exposure was COVID-19 positivity, defined by a positive PCR test. For regression models, CKD patients were separated into three groups: patients hospitalized with COVID-19, patients not-hospitalized with COVID-19 and patients without a COVID-19 positive test.

### Outcome variables

Baseline eGFR was calculated using the 2021race-free CKD-EPI collaboration Eq^37^. as the mean of all eGFR values 12 months (7 to 365 days) preceding the index date. Approximately half of the cohort (51.2%) had at least 2 eGFR values in this timeframe. If there were no values within this timeframe, the mean of all eGFR values 24 months prior to index date was used.

CKD was defined as 2 consecutive eGFR readings < 60 mL/min/1.73m^2^ at least 90 days apart before a diagnosis of COVID-19 or the index date. CKD staging was defined using KDIGO guidelines as G1 (GFR ≥ 90 mL/min/1.73m^2^), G2 (GFR 60–89 mL/min/1.73m^2^), G3a (45–59 mL/min/1.73m^2^), G3b (30–44 mL/min/1.73m^2^), G4 (15–29 mL/min/1.73m^2^), and G5 (< 15 mL/min/1.73m^2^ or initiation of dialysis).

Progression of CKD was defined by advancing to a higher stage of CKD (e.g., those with CKD Stage 3a or 3b progressing to Stage 4, etc.) and was determined at 6, 12, and 24 months using outpatient eGFR measurements. Major adverse kidney event (MAKE) was defined as a composite of eGFR decline of ≥ 30% from baseline or progression to end-stage kidney disease (defined by either an eGFR < 15 mL/min/1.73m^2^ or dialysis dependence). Differences in the annual rate of eGFR change between the groups over 24 month follow up were also examined.

MACE was defined as the composite of all-cause mortality, nonfatal stroke, nonfatal myocardial infarction and non-fatal heart failure using ICD-10 codes.

### Statistical analysis

All statistical analyses were performed using Python packages Statsmodels and lifeline. Frequencies and percentages for categorical variables between the 3 groups of were compared using χ^2^ tests. Continuous variables, expressed as means ± standard deviations, were compared between groups using ANOVA. P-values < 0.05 were considered statistically significant unless otherwise specified.

Multivariable competing risk regression (Fine gray analysis) was used to estimate the hazard ratio (HR) and 95% confidence interval (CI) of progression of CKD to Stage 4 or 5 CKD after COVID-19 at three timepoints (6, 12 and 24 months) with death as a competing risk factor. Patients were censored at death or their last follow up within the health system. Models were adjusted for demographics, comorbidities, AKI, and baseline eGFR.

Incidence of MAKE and MACE were estimated using Kaplan–Meier curves and log-rank hazard ratios (HR) with 95% CI. Cumulative incidence functions were calculated, and Multivariable Cox proportional hazards model was used to estimate HR and 95% CI, adjusting for demographics, comorbidities and baseline eGFR. Patients were censored at death or their last follow up within the health system. Differences between cumulative incidence functions were evaluated using log-rank test.

Linear mixed effects models were used to determine annual eGFR change across the entire follow up period (up to 24 months) for each group. The eGFR is treated as the dependent variable. Time from index date, COVID-19 status, and their interaction were included as the primary factors. Demographics, comorbidities, AKI, and eGFR baseline were also included in the model as covariates. A variance–covariance matrix was used to account for within-subject dependence over time, with model information criteria evaluated to select the optimal matrix structure.

## Results

### Baseline characteristics

**Fig. 1:**
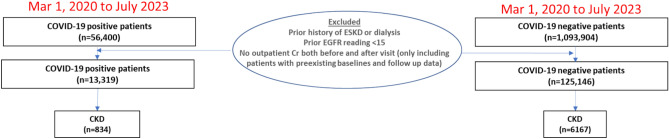
Flowchart of patient selection. From March 11, 2020 to July 1, 2023, there were a total of 56,400 COVID-19 and 1,093,904 non-COVID patients. Abbreviations: CKD, chronic kidney disease. ESKD, end stage kidney disease.

There were 56,400 patients with COVID-19 and 1,093,904 patients without COVID-19 in the Montefiore Health System from March 11, 2020, to July 1, 2023. After exclusions, there were a total of 13,319 COVID-19 positive and 125,146 COVID-19 negative patients (Fig. 1). Of the COVID-19 positive patients, 834 (6.3%) had pre-existing CKD. Of the COVID-19 negative patients, 6,167 (4.9%) had pre-existing CKD.

Table 1 summarizes the demographics and comorbidities grouped by COVID-19 status. CKD patients with COVID-19 were younger, a higher proportion were Hispanics, and a lower proportion were Black compared to those without COVID-19 (p < 0.05). Sex distribution was similar between groups. CKD patients with COVID-19, had higher prevalences of hypertension, diabetes, COPD, asthma, liver disease, heart failure, cancer, and obesity compared to CKD patients without COVID-19. CKD patients with COVID-19 had a lower but similar baseline eGFR (45.1 mL/min/1.73m^2^ ± 9.6 vs 46.5 mL/min/1.73m^2^ ± 9.2). Sixty-four percent of CKD patients with COVID-19 were hospitalized due to COVID-19 and 1.3% of CKD patients with COVID-19 had acute critical illness due to COVID-19. There was no statistical difference in mortality between CKD patients with and without COVID-19 (3.1% vs 2.3%, p = 0.12).

**Table 1:** Characteristics of CKD patients at index date with and without COVID-19. COPD, chronic obstructive pulmonary disorder. COVID-19 critical illness included patients treated in intensive care unit and/or with invasive mechanical ventilation.

	COVID + (n = 834)	COVID- (n = 6167)	P Value
**Demographics**			
Age in years, mean (sd)	75.5 (12.1)	76.9 (11.6)	< 0.001
Male sex, n (%)	321 (38.5%)	2160 (35.0%)	0.05
Hispanic	309 (37.1%)	1815 (29.4%)	< 0.001
White	79 (9.5%)	650 (10.5%)	0.34
Black	353 (42.3%)	2979 (48.3%)	0.001
Other	93 (11.2%)	723 (11.7%)	0.63
**Comorbidities, n (%)**			
Hypertension	801 (96.0%)	5598 (90.8%)	< 0.001
Diabetes	557 (66.8%)	3256 (52.8%)	< 0.001
COPD	150 (18.0%)	757 (12.3%)	< 0.001
Asthma	236 (28.3%)	1058 (17.2%)	< 0.001
Liver	208 (24.9%)	995 (16.1%)	< 0.001
Smoking	245 (29.4%)	1600 (25.9%)	0.04
Heart Failure	246 (29.5%)	1034 (16.8%)	< 0.001
Cancer	192 (23.0%)	1109 (18.0%)	< 0.001
Obesity	318 (38.1%)	1868 (30.3%)	< 0.001
Baseline eGFR	45.1 (9.6)	46.5 (9.2)	< 0.001
AKI	110 (13.2%)	50 (0.8%)	< 0.001
COVID-19 Hospitalization	533 (63.9%)	-	-
COVID-19 Critical Illness	11 (1.3%)	-	-

### Risk of progression to a higher CKD stage

**Fig. 2:**
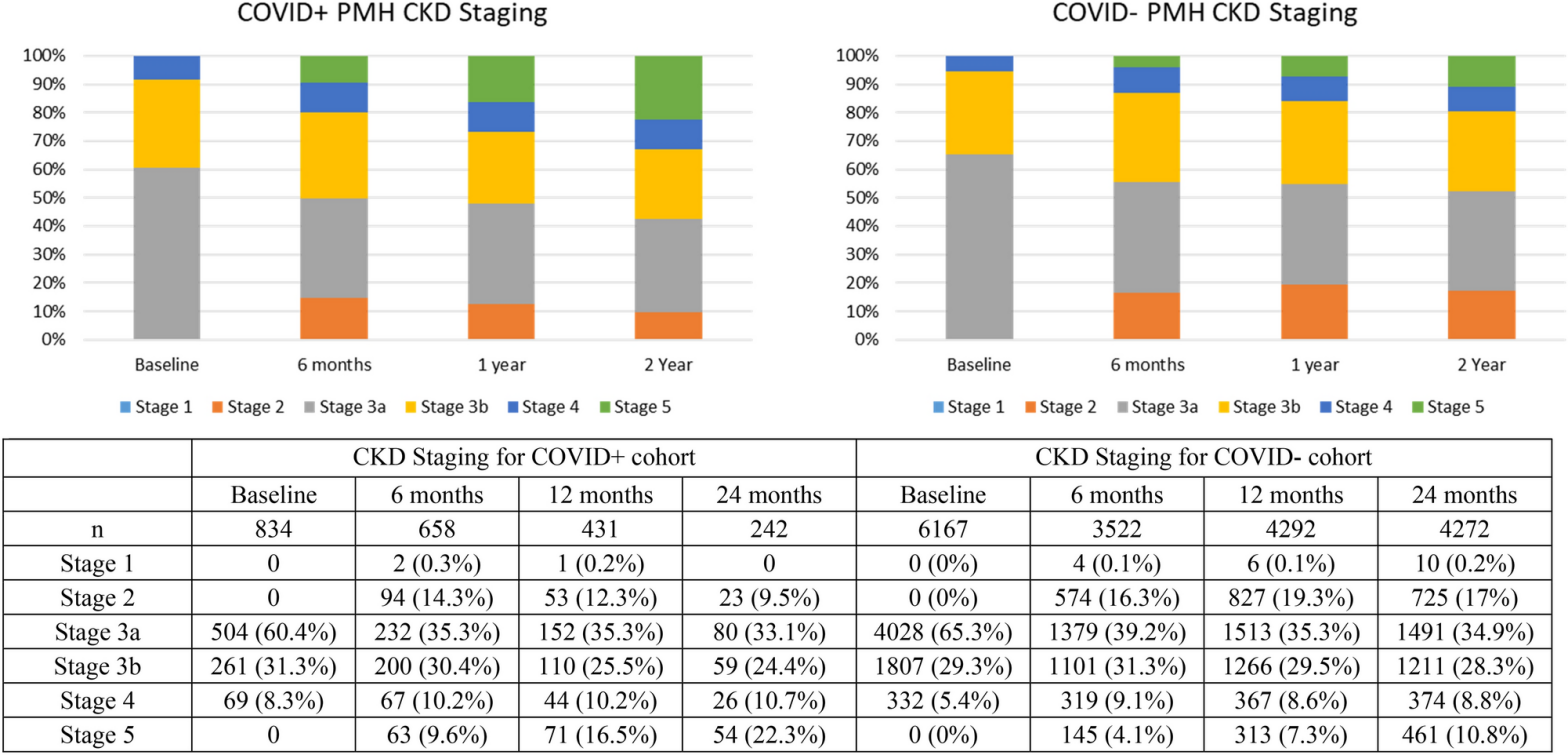
CKD staging as defined by KDIGO guidelines at baseline, 6-, 12- and 24-months post COVID-19 test or index date. PMH, prior medical history. Patients without a COVID-19 positive PCR test was denoted as COVID-.

Figure 2 shows CKD stages at baseline, 6-, 12- and 24-months post index date. A higher proportion of CKD patients with COVID-19 progressed to a more advanced CKD stage compared to those without COVID-19. The number of outpatient eGFR measurements after the index date was similar among CKD patients with versus without COVID-19 (5.0 ± 5.0 vs 4.8 ± 6.1, p = 0.13).

Table 2 shows the results of multivariable regression analysis for progression to Stage 4 or 5 CKD at 6-, 12- and 24-months post index date. CKD patients hospitalized with COVID-19 were not at increased risk of progression to a more advanced CKD stage at 6 months (aHR 1.05 [0.78, 1.42]), but were at increased risk of CKD progression at 12 and 24 months (aHR 1.62 [1.24,2.13], and 1.76 [1.30, 2.40], respectively), compared to non-COVID-19 patients. There was no difference in progression to a more advanced CKD stage among non-hospitalized COVID-19 versus non-COVID-19 patients. The adjusted HRs for covariates for CKD progression are detailed in Supplemental Table 1. For reference, univariable regression results for CKD progression are shown in Supplemental Table 2.

**Table 2:** Adjusted hazard ratios and 95% CI for risk of progression to Stage 4 or 5 at 6, 12 and 24 months post index date, stratified by COVID-19 hospitalization status. Non-COVID-19 patients were used as the reference group. Adjusted for all demographics, comorbidities and baseline eGFR.

	6 months	P Value	12 months	P Value	24 months	P Value
Hospitalized COVID-19	1.05 [0.78,1.42]	0.74	1.62 [1.24,2.13]	< 0.001	1.76 [1.30,2.40]	< 0.001
Non-hospitalized COVID-19	1.21 [0.82,1.78]	0.34	1.43 [0.96,2.14]	0.08	1.49 [0.82,2.71]	0.19

To explore the contribution of AKI to outcomes, multivariable regression analysis for CKD progression excluding patients with AKI was performed (Supplemental Table 3). The overall findings are consistent with those reported above. Among the non-AKI cohort, CKD patients hospitalized with COVID-19 were not at increased risk of progression to a higher CKD stage at 6 months (aHR 1.23 [0.89, 1.69]) but were at increased risk of CKD progression at 12 and 24 months (aHR 1.72 [1.29,2.30] and 2.04 [1.50, 2.78], respectively) compared to non-COVID-19 patients. There were no differences in progression to a higher CKD stage between non-hospitalized COVID-19 and non-COVID-19 patients who did not develop AKI.

**Table 3:** Adjusted hazard ratios (95% CI) for risk factors associated with MAKE at 6-, 12- and 24-months post index date. Non-COVID-19 patients were used as the reference group. Adjusted for all demographics, comorbidities and baseline eGFR.

	6 Months	P Value	1 Year	P Value	2 Year	P Value
Hospitalized COVID-19	2.94 [2.28,3.79]	< 0.001	2.24 [1.81,2.76]	< 0.001	2.03 [1.70,2.42]	< 0.001
Non-hospitalized COVID-19	1.92 [1.32,2.80]	< 0.001	1.81 [1.36,2.42]	< 0.001	1.53 [1.18,1.98]	< 0.001
**Demographics**						
Age	0.99 [0.98,1.00]	0.02	0.99 [0.98,0.99]	< 0.001	0.99 [0.99,0.99]	< 0.001
Male sex	1.09 [0.90,1.32]	0.37	1.15 [1.00,1.32]	0.06	1.14 [1.03,1.27]	0.01
Ethnicity	1.09 [0.85,1.40]	0.49	1.20 [0.99,1.44]	0.06	1.17 [1.02,1.35]	0.02
Black Race	1.18 [0.93,1.49]	0.17	1.19 [0.99,1.41]	0.06	1.09 [0.96,1.24]	0.18
**Comorbidities**						
Hypertension	2.25 [1.31,3.86]	0.003	1.58 [1.13,2.21]	0.01	1.28 [1.02,1.59]	0.03
Diabetes	1.19 [0.98,1.45]	0.08	1.29 [1.12,1.50]	< 0.001	1.34 [1.21,1.50]	< 0.001
COPD	1.15 [0.89,1.49]	0.29	1.13 [0.93,1.37]	0.23	1.16 [1.00,1.35]	0.05
Asthma	1.01 [0.80,1.27]	0.95	1.00 [0.84,1.20]	0.99	1.03 [0.90,1.18]	0.65
Liver	1.46 [1.19,1.80]	< 0.001	1.32 [1.13,1.55]	< 0.001	1.24 [1.10,1.40]	< 0.001
Smoking	1.54 [1.26,1.89]	< 0.001	1.42 [1.22,1.66]	< 0.001	1.24 [1.10,1.39]	< 0.001
Heart Failure	1.33 [1.08,1.65]	0.01	1.46 [1.25,1.71]	< 0.001	1.39 [1.23,1.57]	< 0.001
Cancer	1.30 [1.05,1.61]	0.02	1.21 [1.02,1.42]	0.03	1.28 [1.13,1.44]	< 0.001
Obesity	0.89 [0.73,1.09]	0.25	0.89 [0.76,1.03]	0.11	0.97 [0.87,1.09]	0.64
Baseline eGFR	0.98 [0.97,0.98]	< 0.001	0.97 [0.96,0.98]	< 0.001	0.97 [0.96,0.97]	< 0.001
AKI	5.42 [4.02,7.31]	< 0.001	4.02 [3.07,5.25]	< 0.001	3.05 [2.39,3.89]	< 0.001

### Risk of MAKE

**Fig. 3:**
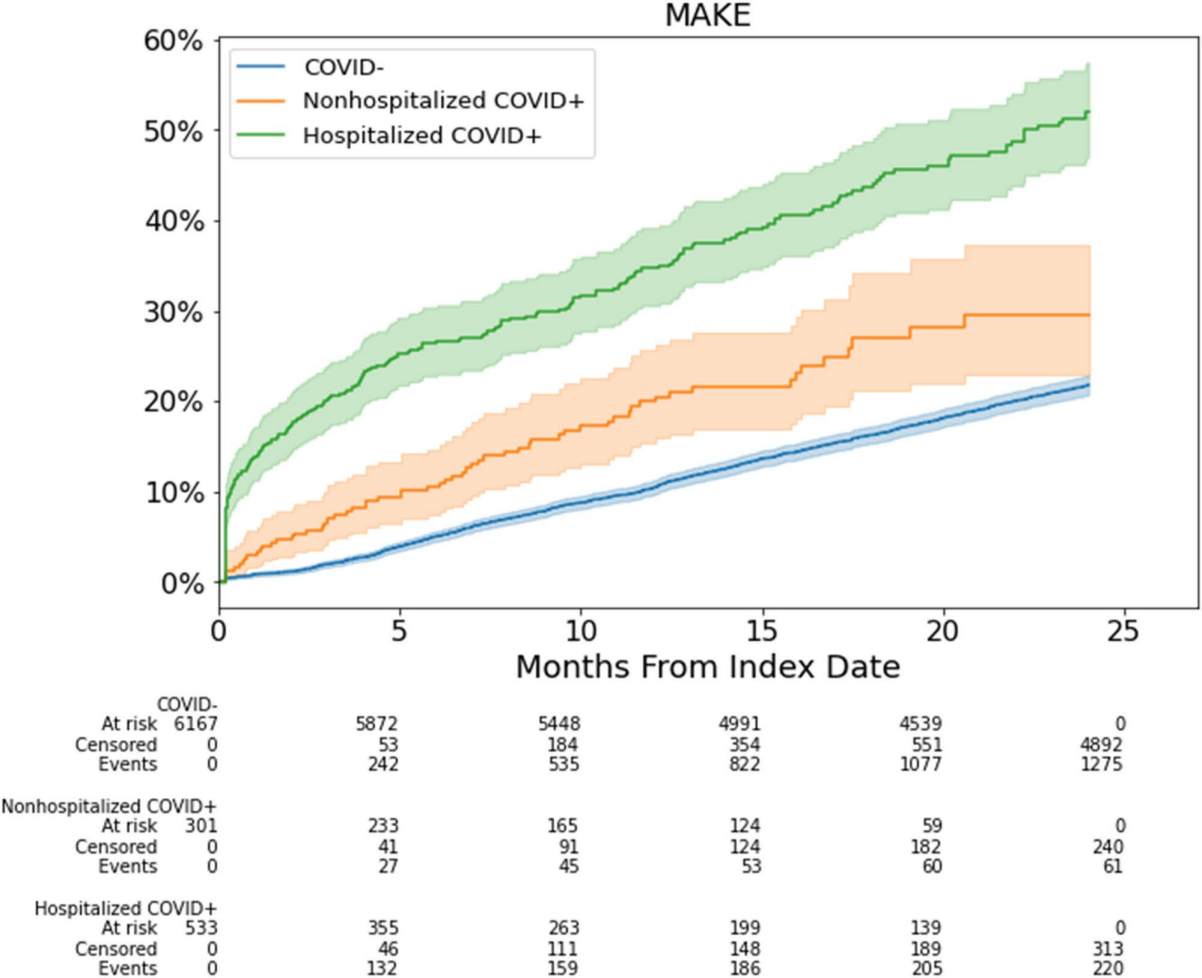
MAKE cumulative incidence curves for CKD patients hospitalized with COVID-19, patients non-hospitalized with COVID-19, and non-COVID-19 (COVID-) patients across the 24 months follow-up period.

Figure 3 shows the cumulative incidence of MAKE stratified by COVID-19 hospitalization status. The median follow-up time was 24 months. CKD patients hospitalized for COVID-19 had a higher incidence of MAKE compared to non-hospitalized CKD patients with COVID-19 (p < 0.001) and non-COVID-19 patients (p < 0.001). Non-hospitalized COVID-19 also had a higher incidence of MAKE compared to non-COVID-19 patients (p < 0.001).

Table 3 shows the multivariable regression analysis for MAKE at 6-, 12- and 24-months. Hospitalized COVID-19 patients were at increased risk of MAKE compared to non-COVID-19 patients at 6-, 12- and 24-months (aHR 2.94 [2.28,3.79], 2.24 [1.81,2.76], and 2.03 [1.70,2.42], respectively). Non-hospitalized COVID-19 patients were also at higher risk compared to non-COVID-19 patients at 6-, 12- and 24-months (aHR 1.90 [1.31,2.78], 1.81 [1.35,2.42]), and 1.53 [1.18,1.99], respectively). Notably, the risk of MAKE decreased over time.

### Annual eGFR decline

CKD patients with COVID-19 experienced a faster annual eGFR decline of −2.12 mL/min/1.73 m^2^ (95%CI [−1.64,2.50], p < 0.001) compared to CKD patients without COVID-19 who had an annual eGFR decline of −1.12 mL/min/1.73 m^2^ (95%CI [−1.01, −1.23], p < 0.001).

### Risk of MACE

**Fig. 4:**
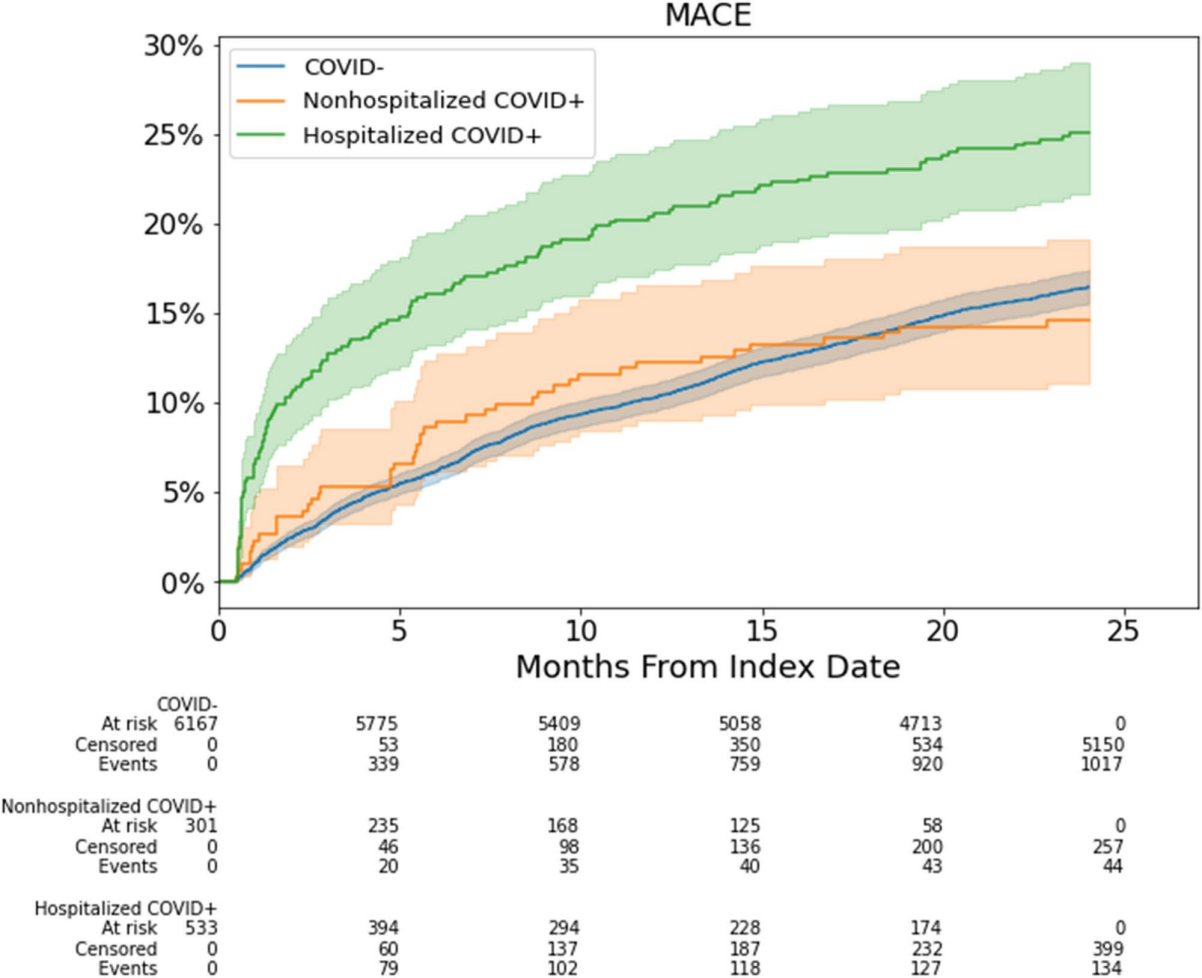
MACE cumulative incidence curves for CKD patients hospitalized with COVID-19, patients non-hospitalized with COVID-19, and non-COVID-19 (COVID-) patients across the 24 months follow-up period.

Figure 4 shows the cumulative incidence of MACE stratified by COVID-19 hospitalization status over a median follow-up time of 24 months. Overall, CKD patients hospitalized for COVID-19 had a higher incidence of MACE compared to non-hospitalized CKD patients with COVID-19 (p < 0.001) and non-COVID-19 patients (p < 0.001). There was no difference in MACE between non-hospitalized COVID-19 versus non-COVID-19 patients (p = 0.47).

Table 4 shows the multivariable regression analysis for MACE at 6-, 12- and 24-months. Hospitalized COVID-19 patients were at increased risk of MACE compared to non-COVID-19 patients at 6-, 12- and 24-months (aHR 1.73 [1.21, 2.50], 1.77 [1.34, 2.33], and 1.31 [1.05, 1.64], respectively) compared to non-COVID-19 patients. Notably, the risk of MACE was highest at 6 and 12 months and decreased over time. Non-hospitalized COVID-19 were at increased risk of MACE compared to non-COVID-19 patients at 6 and 12 months (aHR 1.74 [1.07, 2.83], 1.72 [1.17, 2.53] respectively) but not at 24 months (aHR 1.03 [0.73,1.44]). The adjusted HRs for covariates are detailed in Supplemental Table 4.

**Table 4:** Adjusted hazard ratio and 95% CI for risk of MACE at 6-, 12-, and 24-months post index date, stratified by COVID-19 hospitalization status. COVID-19 negative cohort was used as the reference. Hazard ratios were adjusted for all demographics, comorbidities and eGFR baseline.

	6 months	P Value	12 months	P Value	24 months	P Value
Hospitalized COVID-19	1.73 [1.21,2.50]	< 0.001	1.77 [1.34,2.33]	< 0.001	1.31 [1.05,1.64]	0.02
Non-hospitalized COVID-19	1.74 [1.07,2.83]	0.02	1.72 [1.17,2.53]	0.01	1.03 [0.73,1.44]	0.89

### Hospitalization duration on outcomes

There were 31.1% (166/533) of hospitalized COVID-19 patients hospitalized for 7 or more days. Supplemental Table 5 shows the impact of hospitalization duration on MACE and MAKE. COVID-19 patients hospitalized for 7 or more days were at increased risk of MAKE compared to COVID-19 patients hospitalized for less than 7 days at 6-, 12- and 24-months (aHR 1.86 [1.45,2.39], 1.94 [1.51,2.49], and 2.00 [1.55,2.58], respectively). COVID-19 patients hospitalized for 7 or more days were at increased risk of MACE compared to COVID-19 patients hospitalized for less than 7 days at 6-,12- and 24-months (aHR 1.94 [1.25,3.01], 1.95 [1.32,2.89], and 1.88 [1.32,2.67], respectively).

## Discussion

This study examined the long-term outcomes of patients with CKD up to 24 months after COVID-19 in a diverse population in the Bronx that was hit hard by early pandemic and subsequent surges. Only hospitalized COVID-19 patients with CKD had increased risk of CKD progression and MACE compared to those without COVID-19. Both hospitalized and non-hospitalized COVID-19 patients with CKD had higher risk of MAKE compared to those without COVID-19. To our knowledge, this is the first study to investigate long-term kidney and cardiovascular outcomes after COVID-19 among hospitalized and non-hospitalized non-dialysis CKD patients.

The risk of progression to a more advanced CKD stage among patients hospitalized with COVID-19 was larger or comparable with other CKD risk factors, underscoring the relatively high risk of COVID-19 had on CKD progression even after adjustment for AKI. These observations suggest that the severity of COVID-19 disease contributes to CKD progression. Consistent with the literature, baseline eGFR, hypertension, diabetes, smoking, and heart failure were independently associated with CKD progression^38^.

Numerous studies have reported kidney outcomes after hospitalized patients with COVID-19 were discharged. Among 1,726,683 patients from the Saint Louis VA system, COVID-19 survivors had a 1.62 higher risk of > 50% eGFR decline at 6 months across non-hospitalized, hospitalized, and ICU patients^39^. A multicenter study of 2,212 patients with long COVID-19 symptoms for more than 3 months in British Columbia, Canada observed that the overall cohort had a 3.39% reduction from baseline eGFR within a year^40^. Another multicenter study of 12,891 hospitalized patients from the 4CE consortium observed that patients who developed AKI at the time of COVID-19 had persistent elevation of creatinine (> 125% of baseline) 12 months after infection^8^. Other studies have similarly observed that there is incomplete recovery of kidney function up to 16 months after infection in patients with COVID-19 who developed AKI^22,41–43^. In comparison, Aklilu et al. reported that survivors of hospitalization with COVID-AKI experience lower rates of MAKE and mortality compared with patients with AKI associated with other illnesses (including influenza). The authors speculated that those who survive a COVID-19 complicated by AKI may have inherent unmeasured characteristics that are associated with favorable longer-term outcomes. While we observed a higher risk of MAKE among patients with COVID-19 compared to those without COVID-19, this may be because our cohort that was composed only of patients with pre-existing CKD who are a subgroup at higher risk of rapid kidney function decline compared to those with higher eGFR levels.

CKD progression may be due to persistent activation of the immune system and inflammation following COVID-19^44–48^. Inflammation is a known risk factor for incident and progressive CKD. We surmise that systemic inflammation as a consequence of COVID-19 promotes glomerulosclerosis and tubulointerstitial fibrosis^49^. Though unproven, direct kidney injury may also result from viral infection of the kidney or precipitation of immune-mediated glomerulonephritis^50^.

The risk for MACE in CKD patients hospitalized for COVID-19 attenuated over time suggesting that the contribution of COVID-19 as a risk factor decreased over time. A higher incidence of cardiovascular events has been reported following other respiratory infections. For example, influenza has been associated with an elevated risk of myocardial infarction and stroke, especially within the first few weeks after infection^51–53^. Systemic inflammation as a result of viral infection promotes endothelial dysfunction and destabilizes atherosclerotic plaques, making them prone to rupture, resulting in acute myocardial infarction or stroke^54,55^. Moreover, respiratory infections can alter autonomic nervous system function, leading to increased sympathetic activity, tachycardia and hypertension. These changes increase oxygen demand and can precipitate acute coronary syndrome^56^. Viral-associated inflammation and immune activation also causes a hypercoagulable state, increasing the risk of thrombotic events^57^. This underscores the need for close follow up after viral infections in patients with cardiometabolic risk factors for therapies aimed at cardiovascular risk reduction.

### Pandemic circumstance

Finally, it is important to note that, beyond the direct or indirect effects of COVID-19 on the outcomes described above, the social impact in the early pandemic—such as psychological stress, unhealthy diet, interrupted care, and limited access to healthcare—could also contribute to CKD progression and cardiovascular events^58–60^. Populations with lower socioeconomic status, like ours in an inner-city setting, may be particularly vulnerable to these factors, although this hypothesis was beyond the scope of our study to test.

### Strengths and limitations

Strengths of our study include the long follow-up time of a large diverse cohort, time to event analyses for risk of MAKE and MACE. Our results were consistent across sensitivity analyses for CKD patients with and without AKI.

However, there are notable limitations. First, this is a single health system study. Larger, multi-center studies are needed to improve generalizability. Second, vaccine status, which could affect outcomes, was not reliably recorded and therefore vaccine status was not analyzed^61^. Third, albuminuria is an important predictor of CKD progression but only 14% of our cohort had albuminuria data so we did were unable to use this as a covariate in our models. Fourth, since this is an observational study with real-world data, there was a lack of standardized follow up, tracking of exposures after discharge and our findings are limited to patients who returned to our health system. Although patient records included those who returned for any medical reason, including but not limited to routine office visits, patients who came to our health system may have had more severe COVID-19 and thus might not be representative of the general population at large. Fifth, we only considered the first COVID positive test and did not investigate the effects of multiple infections. COVID-19 status could be misclassified if patients were tested positive elsewhere but did not register in our health system. Sixth, we did not analyze outcomes with respect to various treatments. This was because each patient could have undergone many different treatments and our sample size did not have the power to adjust for these covariates.

## Conclusions

Patients with underlying CKD who are hospitalized for COVID-19 are at increased risk of poor long-term adverse kidney and cardiovascular outcomes. These findings highlight the importance of comprehensive follow-up care to slow CKD progression and prevent cardiovascular events in this high-risk subgroup.

## Supplementary Information

Supplementary Information 1.

Supplementary Information 2.

Supplementary Information 3.

Supplementary Information 4.

Supplementary Information 5.

## Data Availability

Data used is available from the corresponding author upon reasonable request.
