# Supplementary material for: Long term outcomes of patients with chronic kidney disease after COVID-19 in an urban population in the Bronx
Source: Sci Rep. 2025 Feb 19;15:6119. doi: 10.1038/s41598-025-90153-6 (PMC11839904; doi:10.1038/s41598-025-90153-6)
Supplement: Supplementary file 1 — Supplementary Information 1. [file 41598_2025_90153_MOESM1_ESM.docx]

**Supplemental Table 1.** Adjusted hazard ratios for risk of progression to Stage 4 or 5 from baseline eGFR status at 6, 12 and 24 months post the index date. Non-COVID patients were used reference. Note that baseline eGFR and hypertension were significantly associated with progression at all three timepoints (p<0.05). Diabetes, smoking, and heart failure were significantly associated with CKD stage progression at most timepoints (p<0.05). Patients not hospitalized for COVID-19 did not show significant association with progression (p>0.05). Demographic variables also did not show significant association with progression (p>0.05).

|  | 6 Months | P Value | 12-months | P Value | 24 months | P Value |
| --- | --- | --- | --- | --- | --- | --- |
| Hospitalized COVID-19 | 1.05 [0.78,1.42] | 0.74 | 1.62 [1.24,2.13] | <0.001 | 1.76 [1.30,2.40] | <0.001 |
| Non-hospitalized COVID-19 | 1.21 [0.82,1.78] | 0.34 | 1.43 [0.96,2.14] | 0.08 | 1.49 [0.82,2.71] | 0.19 |
|  |  |  |  |  |  |  |
| **Demographics** |  |  |  |  |  |  |
| Age | 0.99 [0.98,1.00] | 0.08 | 0.99 [0.98,1.00] | 0.02 | 0.99 [0.99,1.00] | 0.24 |
| Male sex | 1.06 [0.87,1.30] | 0.58 | 1.10 [0.93,1.29] | 0.27 | 1.25 [1.08,1.44] | <0.001 |
| Ethnicity | 1.10 [0.85,1.43] | 0.47 | 1.16 [0.94,1.43] | 0.17 | 1.25 [1.03,1.51] | 0.02 |
| Black Race | 1.27 [0.99,1.61] | 0.05 | 1.08 [0.88,1.31] | 0.46 | 1.17 [0.98,1.40] | 0.09 |
|  |  |  |  |  |  |  |
| **Comorbidities** |  |  |  |  |  |  |
| Hypertension | 1.88 [1.07,3.30] | 0.03 | 1.77 [1.15,2.73] | 0.01 | 1.41 [1.01,1.96] | 0.04 |
| Diabetes | 1.35 [1.10,1.67] | <0.001 | 1.28 [1.08,1.51] | <0.001 | 1.13 [0.98,1.31] | 0.09 |
| COPD | 1.30 [0.98,1.71] | 0.06 | 1.15 [0.91,1.45] | 0.25 | 1.27 [1.04,1.57] | 0.02 |
| Asthma | 0.86 [0.66,1.11] | 0.24 | 0.96 [0.78,1.18] | 0.69 | 0.95 [0.79,1.14] | 0.56 |
| Liver | 1.23 [0.98,1.54] | 0.07 | 1.08 [0.88,1.31] | 0.46 | 1.15 [0.97,1.37] | 0.12 |
| Smoking | 1.25 [1.01,1.55] | 0.04 | 1.16 [0.97,1.39] | 0.10 | 1.26 [1.08,1.47] | <0.001 |
| Heart Failure | 1.17 [0.94,1.46] | 0.15 | 1.40 [1.17,1.67] | <0.001 | 1.20 [1.02,1.42] | 0.03 |
| Cancer | 1.02 [0.80,1.29] | 0.87 | 1.07 [0.88,1.30] | 0.50 | 1.03 [0.87,1.23] | 0.71 |
| Obesity | 1.00 [0.81,1.23] | 0.99 | 1.03 [0.87,1.21] | 0.76 | 1.05 [0.90,1.22] | 0.55 |
| Baseline eGFR | 0.93 [0.92,0.94] | <0.001 | 0.93 [0.93,0.94] | <0.001 | 0.93 [0.93,0.94] | <0.001 |
| AKI | 4.11 [2.89,5.84] | <0.001 | 2.58 [1.78,3.73] | <0.001 | 1.75 [1.15,2.67] | 0.01 |
