# Supplementary material for: Long term outcomes of patients with chronic kidney disease after COVID-19 in an urban population in the Bronx
Source: Sci Rep. 2025 Feb 19;15:6119. doi: 10.1038/s41598-025-90153-6 (PMC11839904; doi:10.1038/s41598-025-90153-6)
Supplement: Supplementary file 2 — Supplementary Information 2. [file 41598_2025_90153_MOESM2_ESM.docx]

**Supplemental Table 2.** Univariable Regression for progression to Stage 4 or 5 CKD at 6-, 12- and 24-months post COVID-19 test or index date. eGFR baseline, COVID-19 hospitalization, hypertension, diabetes, smoking, and heart failure were significantly associated with worsening CKD at all three timepoints (p<0.05).

|  | 6 months | P Value | 12 months | P Value | 24 months | P Value |
| --- | --- | --- | --- | --- | --- | --- |
| Hospitalized COVID-19 | 2.08 [1.61,2.68] | <0.001 | 2.51 [1.97,3.18] | <0.001 | 2.52 [1.95,3.24] | <0.001 |
| Non-hospitalized COVID-19 | 1.24 [0.85,1.82] | 0.27 | 1.36 [0.91,2.03] | 0.13 | 1.21 [0.67,2.19] | 0.54 |
|  |  |  |  |  |  |  |
| **Demographics** |  |  |  |  |  |  |
| Age | 0.99 [0.99,1.00] | 0.07 | 0.99 [0.99,1.00] | 0.04 | 0.99 [0.98,1.00] | 0.001 |
| Male sex | 1.16 [0.96,1.41] | 0.13 | 1.17 [1.00,1.37] | 0.04 | 1.33 [1.15,1.52] | <0.001 |
| Ethnicity | 0.88 [0.69,1.13] | 0.31 | 1.24 [1.02,1.51] | 0.03 | 1.19 [1.00,1.43] | 0.05 |
| Black Race | 1.24 [1.03,1.49] | 0.03 | 0.97 [0.84,1.13] | 0.72 | 1.04 [0.91,1.19] | 0.56 |
|  |  |  |  |  |  |  |
| **Comorbidities** |  |  |  |  |  |  |
| Hypertension | 3.03 [1.75,5.26] | <0.001 | 2.65 [1.73,4.06] | <0.001 | 1.91 [1.39,2.64] | <0.001 |
| Diabetes | 1.75 [1.43,2.14] | <0.001 | 1.68 [1.42,1.97] | 0.00 | 1.36 [1.18,1.56] | <0.001 |
| COPD | 1.43 [1.12,1.83] | <0.001 | 1.35 [1.10,1.66] | <0.001 | 1.41 [1.18,1.70] | <0.001 |
| Asthma | 0.97 [0.77,1.23] | 0.81 | 1.09 [0.90,1.32] | 0.38 | 1.05 [0.88,1.24] | 0.59 |
| Liver | 1.36 [1.10,1.70] | 0.01 | 1.15 [0.94,1.39] | 0.17 | 1.17 [0.98,1.39] | 0.08 |
| Smoking | 1.49 [1.22,1.81] | <0.001 | 1.31 [1.11,1.54] | 0.001 | 1.48 [1.28,1.71] | <0.001 |
| Heart Failure | 1.79 [1.46,2.20] | <0.001 | 1.93 [1.63,2.28] | <0.001 | 1.64 [1.40,1.93] | <0.001 |
| Cancer | 1.06 [0.84,1.33] | 0.64 | 1.06 [0.87,1.28] | 0.57 | 1.09 [0.92,1.29] | 0.33 |
| Obesity | 1.27 [1.04,1.54] | 0.02 | 1.27 [1.08,1.48] | 0.003 | 1.20 [1.04,1.38] | 0.01 |
| Baseline eGFR | 0.92 [0.92,0.93] | <0.001 | 0.93 [0.92,0.94] | <0.001 | 0.93 [0.93,0.94] | <0.001 |
| AKI | 5.98 [4.42,8.09] | <0.001 | 4.66 [3.36,6.46] | <0.001 | 3.56 [2.51,5.04] | <0.001 |
