# Supplementary material for: Long term outcomes of patients with chronic kidney disease after COVID-19 in an urban population in the Bronx
Source: Sci Rep. 2025 Feb 19;15:6119. doi: 10.1038/s41598-025-90153-6 (PMC11839904; doi:10.1038/s41598-025-90153-6)
Supplement: Supplementary file 3 — Supplementary Information 3. [file 41598_2025_90153_MOESM3_ESM.docx]

**Supplemental Table 3.** Adjusted hazard ratios for risk of progression to Stage 4 or 5 from baseline eGFR status at 6, 12 and 24 months post the index date in patients who did not have AKI. Non-COVID patients were used as the reference. Non-hospitalized COVID-19 patients were not at increased risk of CKD progression (p>0.05). Demographic variables also did not show independent associations with CKD progression (p>0.05).

|  | 6 Months | P Value | 1 Year | P Value | 2 Year | P Value |
| --- | --- | --- | --- | --- | --- | --- |
| Hospitalized COVID-19 | 1.23 [0.89,1.69] | 0.21 | 1.72 [1.29,2.30] | <0.001 | 2.04 [1.50,2.78] | <0.001 |
| Non-hospitalized COVID-19 | 1.33 [0.89,2.00] | 0.16 | 1.41 [0.93,2.12] | 0.10 | 1.51 [0.83,2.74] | 0.18 |
|  |  |  |  |  |  |  |
| **Demographics** |  |  |  |  |  |  |
| Age | 0.99 [0.98,1.00] | 0.09 | 0.99 [0.99,1.00] | 0.06 | 0.99 [0.98,1.00] | <0.001 |
| Male sex | 1.12 [0.91,1.39] | 0.29 | 1.11 [0.94,1.31] | 0.22 | 1.28 [1.11,1.49] | <0.001 |
| Ethnicity | 1.16 [0.88,1.53] | 0.28 | 1.17 [0.94,1.45] | 0.16 | 1.25 [1.02,1.51] | 0.03 |
| Black Race | 1.31 [1.01,1.68] | 0.04 | 1.08 [0.89,1.33] | 0.44 | 1.16 [0.97,1.39] | 0.11 |
|  |  |  |  |  |  |  |
| **Comorbidities** |  |  |  |  |  |  |
| Hypertension | 1.80 [1.02,3.16] | 0.04 | 1.67 [1.08,2.58] | 0.02 | 1.39 [1.00,1.94] | 0.05 |
| Diabetes | 1.28 [1.02,1.60] | 0.03 | 1.26 [1.06,1.50] | 0.01 | 1.12 [0.96,1.29] | 0.15 |
| COPD | 1.47 [1.10,1.96] | 0.01 | 1.19 [0.93,1.51] | 0.16 | 1.26 [1.02,1.56] | 0.03 |
| Asthma | 0.74 [0.56,0.99] | 0.04 | 0.89 [0.72,1.11] | 0.31 | 0.90 [0.74,1.09] | 0.26 |
| Liver | 1.24 [0.97,1.57] | 0.08 | 1.06 [0.86,1.30] | 0.59 | 1.11 [0.93,1.33] | 0.24 |
| Smoking | 1.22 [0.98,1.52] | 0.08 | 1.16 [0.97,1.39] | 0.10 | 1.28 [1.09,1.50] | <0.001 |
| Heart Failure | 1.29 [1.03,1.62] | 0.03 | 1.50 [1.25,1.80] | <0.001 | 1.20 [1.01,1.43] | 0.03 |
| Cancer | 0.98 [0.76,1.26] | 0.88 | 1.05 [0.86,1.28] | 0.65 | 1.06 [0.89,1.26] | 0.54 |
| Obesity | 1.05 [0.85,1.31] | 0.66 | 1.05 [0.89,1.25] | 0.57 | 1.06 [0.91,1.24] | 0.43 |
| Baseline eGFR | 0.93 [0.92,0.93] | <0.001 | 0.93 [0.93,0.94] | <0.001 | 0.93 [0.93,0.94] | <0.001 |
