# Supplementary material for: Long term outcomes of patients with chronic kidney disease after COVID-19 in an urban population in the Bronx
Source: Sci Rep. 2025 Feb 19;15:6119. doi: 10.1038/s41598-025-90153-6 (PMC11839904; doi:10.1038/s41598-025-90153-6)
Supplement: Supplementary file 4 — Supplementary Information 4. [file 41598_2025_90153_MOESM4_ESM.docx]

**Supplemental Table 4.** Adjusted hazard ratios for risk factors associated with MACE at 6-, 12- and 24-months post index date. Non-COVID patients were used reference. Note that baseline eGFR, age and male sex, diabetes were significantly associated with MACE at all three timepoints (p<0.05).

|  | 6 months | P Value | 1 Year | P Value | 2 Year | P Value |
| --- | --- | --- | --- | --- | --- | --- |
| Hospitalized COVID-19 | 1.73 [1.21,2.50] | <0.001 | 1.77 [1.34,2.33] | <0.001 | 1.31 [1.05,1.64] | 0.02 |
| Non-hospitalized COVID-19 | 1.74 [1.07,2.83] | 0.02 | 1.72 [1.17,2.53] | 0.01 | 1.03 [0.73,1.44] | 0.89 |
|  |  |  |  |  |  |  |
| **Demographics** |  |  |  |  |  |  |
| Age | 1.01 [1.00,1.02] | 0.05 | 1.01 [1.01,1.02] | <0.001 | 1.01 [1.01,1.02] | <0.001 |
| Male sex | 1.45 [1.13,1.85] | <0.001 | 1.40 [1.17,1.68] | <0.001 | 1.34 [1.16,1.55] | <0.001 |
| Ethnicity | 0.91 [0.66,1.26] | 0.58 | 1.00 [0.78,1.27] | 0.98 | 1.00 [0.84,1.21] | 0.96 |
| Black Race | 1.00 [0.75,1.34] | 0.99 | 1.10 [0.88,1.37] | 0.39 | 1.02 [0.86,1.21] | 0.79 |
|  |  |  |  |  |  |  |
| **Comorbidities** |  |  |  |  |  |  |
| Hypertension | 1.55 [0.86,2.79] | 0.15 | 1.31 [0.87,1.98] | 0.19 | 1.2 [0.89,1.62] | 0.23 |
| Diabetes | 1.52 [1.17,1.97] | <0.001 | 1.50 [1.24,1.82] | <0.001 | 1.39 [1.20,1.61] | <0.001 |
| COPD | 1.26 [0.90,1.75] | 0.18 | 1.19 [0.93,1.53] | 0.16 | 1.15 [0.94,1.40] | 0.17 |
| Asthma | 0.78 [0.56,1.09] | 0.14 | 0.97 [0.77,1.23] | 0.8 | 1.02 [0.85,1.22] | 0.86 |
| Liver | 0.90 [0.66,1.24] | 0.53 | 1.00 [0.80,1.26] | 0.99 | 1.01 [0.84,1.20] | 0.94 |
| Smoking | 1.24 [0.95,1.62] | 0.12 | 1.20 [0.98,1.47] | 0.07 | 1.10 [0.94,1.29] | 0.24 |
| Cancer | 2.46 [1.91,3.17] | <0.001 | 2.42 [2.00,2.92] | <0.001 | 2.29 [1.98,2.66] | <0.001 |
| Obesity | 0.69 [0.50,0.96] | 0.03 | 0.90 [0.72,1.13] | 0.36 | 1.00 [0.84,1.19] | 0.99 |
| Baseline eGFR | 0.99 [0.98,1.00] | 0.11 | 0.99 [0.98,1.00] | <0.001 | 0.98 [0.98,0.99] | <0.001 |
| AKI | 2.07 [1.24,3.44] | 0.01 | 1.78 [1.18,2.67] | 0.01 | 1.38 [0.96,1.98] | 0.08 |
