# Supplementary material for: Long term outcomes of patients with chronic kidney disease after COVID-19 in an urban population in the Bronx
Source: Sci Rep. 2025 Feb 19;15:6119. doi: 10.1038/s41598-025-90153-6 (PMC11839904; doi:10.1038/s41598-025-90153-6)
Supplement: Supplementary file 5 — Supplementary Information 5. [file 41598_2025_90153_MOESM5_ESM.docx]

**Supplemental Table 5.** Adjusted hazard ratios for risk factors associated with (A) MAKE and (B) MACE at 6-, 12- and 24-months post index date. COVID patients hospitalized for <7 days were used reference. Note that hypertension was removed from MACE prediction due to multicollinearity with other covariates.

**A)**

|  | 6 Months | P Value | 1 Year | P Value | 2 Year | P Value |
| --- | --- | --- | --- | --- | --- | --- |
| Hospitalized ≥ 7 days | 1.86 [1.45,2.39] | <0.001 | 1.94 [1.51,2.49] | <0.001 | 2.00 [1.55,2.58] | <0.001 |
|  |  |  |  |  |  |  |
| **Demographics** |  |  |  |  |  |  |
| Age | 0.99 [0.98,0.99] | <0.001 | 0.99 [0.98,0.99] | <0.001 | 0.99 [0.98,0.99] | <0.001 |
| Male sex | 1.13 [1.02,1.26] | 0.02 | 1.15 [1.03,1.27] | 0.01 | 1.15 [1.04,1.28] | 0.01 |
| Ethnicity | 1.21 [1.06,1.39] | 0.01 | 1.20 [1.04,1.37] | 0.01 | 1.19 [1.04,1.36] | 0.01 |
| Black Race | 1.10 [0.97,1.26] | 0.13 | 1.10 [0.96,1.25] | 0.16 | 1.09 [0.96,1.24] | 0.20 |
|  |  |  |  |  |  |  |
| **Comorbidities** |  |  |  |  |  |  |
| Hypertension | 1.30 [1.04,1.62] | 0.02 | 1.30 [1.04,1.63] | 0.02 | 1.30 [1.04,1.63] | 0.02 |
| Diabetes | 1.37 [1.23,1.53] | <0.001 | 1.37 [1.23,1.52] | <0.001 | 1.37 [1.23,1.53] | <0.001 |
| COPD | 1.15 [0.99,1.34] | 0.06 | 1.17 [1.01,1.36] | 0.04 | 1.17 [1.01,1.36] | 0.03 |
| Asthma | 1.03 [0.90,1.17] | 0.69 | 1.03 [0.91,1.18] | 0.61 | 1.03 [0.90,1.18] | 0.66 |
| Liver | 1.23 [1.09,1.39] | <0.001 | 1.25 [1.11,1.41] | <0.001 | 1.26 [1.11,1.42] | <0.001 |
| Smoking | 1.32 [1.17,1.49] | <0.001 | 1.30 [1.15,1.47] | <0.001 | 1.30 [1.15,1.46] | <0.001 |
| Heart Failure | 1.38 [1.23,1.55] | <0.001 | 1.42 [1.26,1.60] | <0.001 | 1.42 [1.26,1.60] | <0.001 |
| Cancer | 1.32 [1.17,1.49] | <0.001 | 1.30 [1.15,1.47] | <0.001 | 1.30 [1.15,1.46] | <0.001 |
| Obesity | 0.98 [0.88,1.09] | 0.71 | 0.97 [0.87,1.08] | 0.55 | 0.97 [0.87,1.08] | 0.55 |
| Baseline eGFR | 0.97 [0.96,0.97] | <0.001 | 0.97 [0.96,0.97] | <0.001 | 0.97 [0.96,0.97] | <0.001 |
| AKI | 3.66 [2.91,4.59] | <0.001 | 3.86 [3.07,4.86] | <0.001 | 3.94 [3.12,4.98] | <0.001 |

**B)**

|  | 6 Months | P Value | 1 Year | P Value | 2 Year | P Value |
| --- | --- | --- | --- | --- | --- | --- |
| Hospitalized ≥ 7 days | 1.94 [1.25,3.01] | <0.001 | 1.95 [1.32,2.89] | <0.001 | 1.88 [1.32,2.67] | <0.001 |
|  |  |  |  |  |  |  |
| **Demographics** |  |  |  |  |  |  |
| Age | 1.02 [1.00,1.04] | 0.06 | 1.02 [1.00,1.04] | 0.02 | 1.02 [1.00,1.03] | 0.06 |
| Male sex | 1.97 [1.27,3.05] | <0.001 | 1.57 [1.07,2.31] | 0.02 | 1.43 [1.01,2.03] | 0.05 |
| Ethnicity | 0.73 [0.42,1.26] | 0.26 | 0.83 [0.51,1.35] | 0.46 | 0.79 [0.51,1.23] | 0.30 |
| Black Race | 0.68 [0.39,1.18] | 0.18 | 0.72 [0.44,1.18] | 0.20 | 0.65 [0.42,1.02] | 0.06 |
|  |  |  |  |  |  |  |
| **Comorbidities** |  |  |  |  |  |  |
| Diabetes | 1.51 [0.89,2.54] | 0.13 | 1.30 [0.83,2.02] | 0.25 | 1.23 [0.82,1.83] | 0.31 |
| COPD | 1.19 [0.67,2.11] | 0.56 | 1.08 [0.64,1.81] | 0.77 | 1.09 [0.68,1.74] | 0.73 |
| Asthma | 0.85 [0.50,1.44] | 0.54 | 0.78 [0.49,1.24] | 0.29 | 0.83 [0.55,1.26] | 0.38 |
| Liver | 0.78 [0.47,1.30] | 0.34 | 0.77 [0.49,1.21] | 0.26 | 0.84 [0.56,1.25] | 0.39 |
| Smoking | 1.43 [0.88,2.34] | 0.15 | 1.60 [1.03,2.49] | 0.04 | 1.37 [0.91,2.07] | 0.13 |
| Cancer | 0.66 [0.38,1.14] | 0.14 | 0.70 [0.43,1.13] | 0.14 | 0.65 [0.41,1.02] | 0.06 |
| Obesity | 1.17 [0.74,1.86] | 0.50 | 1.20 [0.80,1.80] | 0.38 | 1.11 [0.77,1.60] | 0.59 |
| Baseline eGFR | 0.99 [0.96,1.01] | 0.25 | 0.99 [0.97,1.01] | 0.57 | 0.99 [0.97,1.00] | 0.14 |
| AKI | 1.34 [0.79,2.27] | 0.29 | 1.45 [0.92,2.29] | 0.11 | 1.34 [0.88,2.04] | 0.17 |
